# Maternal Antibody-Mediated Disease Enhancement in Type I Interferon-Deficient Mice Leads to Lethal Disease Associated with Liver Damage

**DOI:** 10.1371/journal.pntd.0004536

**Published:** 2016-03-23

**Authors:** Julia María Martínez Gómez, Li Ching Ong, Jian Hang Lam, Siti Amanlina Binte Aman, Eshele Anak Libau, Pei Xuan Lee, Ashley L. St. John, Sylvie Alonso

**Affiliations:** 1 Department of Microbiology, Yong Loo Lin School of Medicine, National University of Singapore, Singapore; 2 Immunology programme, Life Sciences Institute, National University of Singapore, Singapore; 3 Infectious Disease programme, Singapore-MIT alliance for Research and Technology (SMART), National University of Singapore, Singapore; 4 Emerging Infectious Diseases programme, Duke-NUS, Singapore; Oregon Health and Science University, UNITED STATES

## Abstract

Epidemiological studies have reported that most of the severe dengue cases occur upon a secondary heterologous infection. Furthermore, babies born to dengue immune mothers are at greater risk of developing severe disease upon primary infection with a heterologous or homologous dengue virus (DENV) serotype when maternal antibodies reach sub-neutralizing concentrations. These observations have been explained by the antibody mediated disease enhancement (ADE) phenomenon whereby heterologous antibodies or sub-neutralizing homologous antibodies bind to but fail to neutralize DENV particles, allowing Fc-receptor mediated entry of the virus-antibody complexes into host cells. This eventually results in enhanced viral replication and heightened inflammatory responses. In an attempt to replicate this ADE phenomenon in a mouse model, we previously reported that upon DENV2 infection 5-week old type I and II interferon (IFN) receptors-deficient mice (AG129) born to DENV1-immune mothers displayed enhancement of disease severity characterized by increased virus titers and extensive vascular leakage which eventually led to the animals’ death. However, as dengue occurs in immune competent individuals, we sought to reproduce this mouse model in a less immunocompromised background. Here, we report an ADE model that is mediated by maternal antibodies in type I IFN receptor-deficient A129 mice. We show that 5-week old A129 mice born to DENV1-immune mothers succumbed to a DENV2 infection within 4 days that was sub-lethal in mice born to naïve mothers. Clinical manifestations included extensive hepatocyte vacuolation, moderate vascular leakage, lymphopenia, and thrombocytopenia. Anti-TNFα therapy totally protected the mice and correlated with healthy hepatocytes. In contrast, blocking IL-6 did not impact the virus titers or disease outcome. This A129 mouse model of ADE may help dissecting the mechanisms involved in dengue pathogenesis and evaluate the efficacy of vaccine and therapeutic candidates.

## Author Summary

Severe dengue has been linked to secondary heterologous infection or to primary infection in 6–9 month old babies born to dengue immune mothers. The leading hypothesis for such observations is the antibody-dependent enhancement (ADE) phenomenon in which cross-reactive, but non-neutralizing, heterologous antibodies, or sub-neutralizing levels of homotypic antibodies, enhance uptake of dengue virus into host cells. We report here the development of a dengue ADE mouse model that is mediated by maternal antibodies acquired during gestation and breastfeeding. Dengue infection of mice born to mothers immune to a heterologous dengue serotype resulted in rapid death of the mice, accompanied by increased virus titers, severe liver damage and heighted systemic inflammation. Anti-TNFα therapy fully rescued the animals. This novel mouse model may help further understand dengue pathogenesis and provides a platform for testing the efficacy of vaccine and therapeutic candidates.

## Introduction

Dengue is the most serious and widespread arthropod borne viral disease worldwide with an estimated 390 million people infected mainly in the tropical and subtropical regions, and 3 billion people at risk of infection in over 100 countries [1]. The etiological agent of dengue, dengue virus (DENV), belongs to the genus Flavivirus within the *Flaviviridae* family, which also includes Japanese encephalitis, West Nile, and yellow fever viruses. DENV is an enveloped virus with a single-stranded, positive-sense RNA genome. There are four antigenically distinct serotypes of DENV (DENV1-4) that may co-circulate in the same geographical area [1]. The virus is primarily transmitted to humans by the highly urbanized *Aedes aegypti* female mosquito which has spread globally due to increased trade and travel [2]. *Aedes albopictus* has also been reported to effectively transmit DENV to humans and its capacity to survive in cooler weather has allowed the spread of the virus to more temperate regions such as Europe and North America [3].

Human infection with one of the four DENV serotypes is mostly asymptomatic. When symptomatic, the disease presents itself in a wide spectrum of clinical manifestations, ranging from mild acute febrile illness to self-limiting classical dengue fever (DF) to the severe dengue haemorrhagic fever/dengue shock syndrome (DHF/ DSS) [4]. The hallmarks of DHF/DSS are haemorrhagic manifestations and increased vascular permeability, respectively, the latter resulting in fluid loss which may progress to life-threatening hypovolemic shock.

While infection with one DENV serotype is believed to confer life-long protection against that particular serotype, secondary infection with a heterologous serotype may lead to severe disease. Epidemiological studies over the last few decades have indeed reported that most of the DHF/DSS cases occur upon secondary infection with a heterologous DENV serotype [5, 6]. Increased risk of DHF/DSS was also reported in infants at 5–9 months of age born to dengue immune mothers when maternally acquired antibodies wane to sub-neutralizing levels [7–10]. These epidemiological observations were explained by the antibody-dependent enhancement (ADE) of infection hypothesis, whereby actively (during primary infection) or passively (through maternal transfer) acquired anti-DENV antibodies cross react but fail to neutralize a heterologous (or homologous) serotype of DENV [5]. Mechanistically, antibody-opsonized DENV gains entry into activating Fc receptor (FcR)-bearing cells such as dendritic cells and monocytes resulting in increased viral replication which in turn triggers the massive release of inflammatory and vasoactive mediators that contribute to the disease severity [11–13].

The lack of suitable animal models for DENV infection has seriously hindered the understanding of dengue pathogenesis and the pre-clinical evaluation of prophylactic and therapeutic candidates. Immunocompetent mice are generally not susceptible to DENV infection partly due to the virus inability to interfere with the murine type I IFN response [14–17]. However, a handful of studies have described that upon intravenous administration of high doses (10^8^ pfu) of DENV to immunocompetent mice, viral load could be detected in the serum and other organs, accompanied with relevant clinical manifestations such as hemorrhage and thrombocytopenia [18, 19]. However, immunocompetent mouse models are seldom used due to the major shortcoming of low/undetectable systemic infective viremia.

Alternatively, a variety of mouse genetic backgrounds lacking various immune components have been explored that displayed increased susceptibility to DENV infection [20–24]. Among these models, AG129 mice, which are deficient in interferon (IFN)-α/β and -γ receptors, were shown by us and others to allow effective replication of a number of DENV strains, mainly from serotype 1 and 2 [23, 25–28]. ADE models have also been reported in these mice whereby administration of cross-reactive, sub- or non-neutralizing antibodies led to enhanced disease severity [29, 30]. We have recently reported that maternally acquired heterotypic dengue antibodies induced ADE in AG129 mice, with earlier death, increased viremia and increased vascular permeability upon DENV2 infection of mice born to DENV1-immune mothers compared to mice born to naïve mothers [31]. While this model is supported by many epidemiological observations, the lack of an intact IFNγ signaling pathway in these mice limits the study of certain aspects of the dynamic interactions between the antibodies, virus particles and immune cells. Indeed, the antibody production in AG129 mice has been reported to be biased towards the IgG1 subclass due to the lack of IFNγ [23]. However in humans, DENV infection results mainly in IgG1 and IgG3 [32] which corresponds in mice to the IgG2a subclass. Consistently, DENV infection of immune competent mice mainly results in the production of IgG2a antibodies [33]. As IgG subclasses display differential affinities for various activating and inhibitory Fc-receptors [11–13], their role and impact on disease severity in the context of ADE is likely to be different. Therefore, we sought to address this aspect by reproducing the maternal ADE model in A129 mice, which are type I IFN-receptor deficient but have an intact IFNγ signaling pathway. We report here that DENV2 infection of young mice born to DENV1-immune mothers resulted in enhanced disease severity characterized by death, increased viremia, and severe liver damage. The detrimental role of TNFα in this model was also demonstrated.

## Results

### Cross-reactive, neutralizing and enhancing properties of sera from DENV1-immune mothers and their offspring

**Fig 1:**
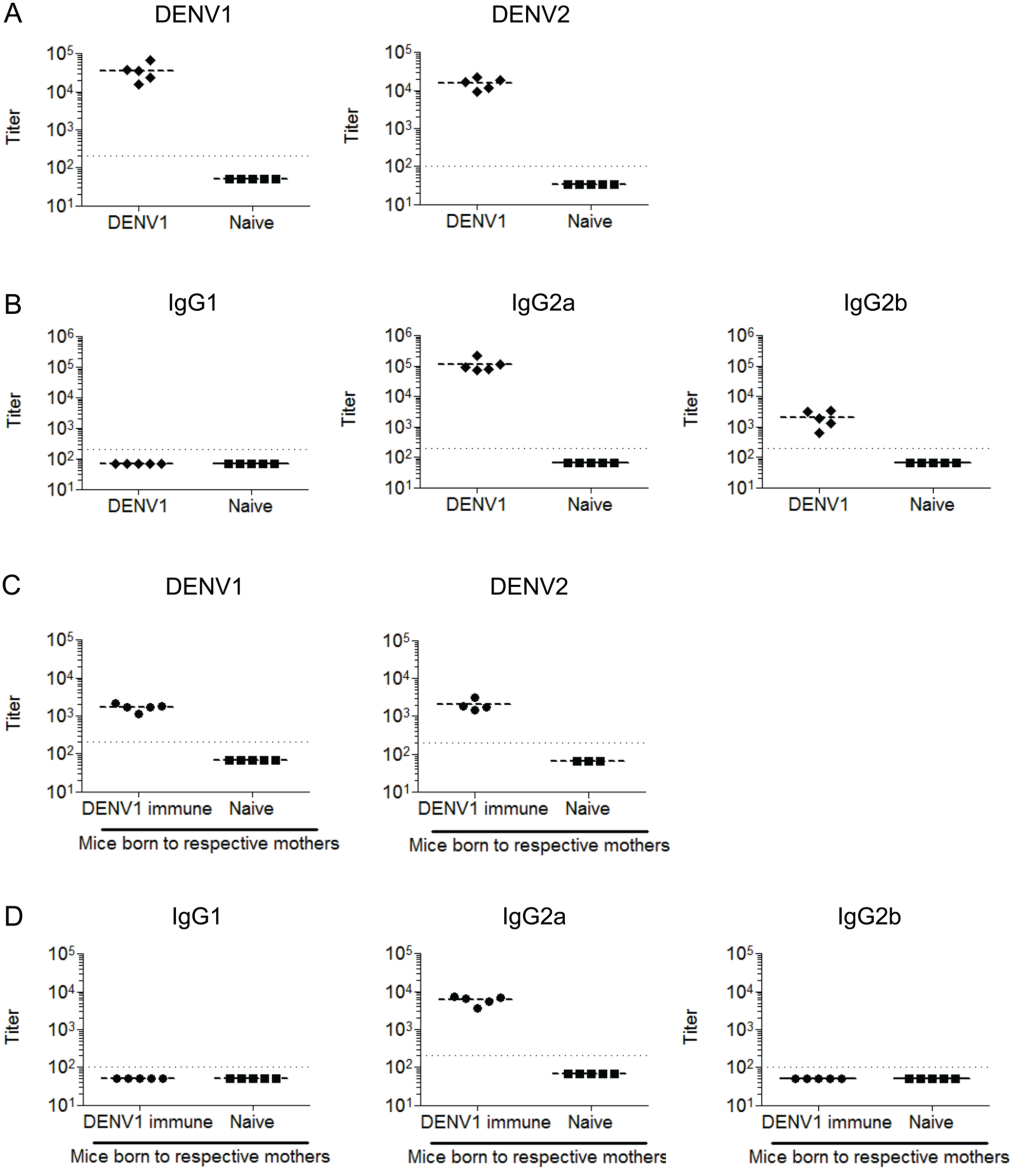
DENV-specific antibody titers in DENV1-immune mothers and their offspring. Five to six weeks old naïve female A129 mice were infected iv with 10^6^ PFU of DENV1 and bled at 6 weeks post-infection (n = 3–5) (A&B). A129 mice born to naïve or DENV1-immune mothers were bled at the age of 5–6 weeks old (n = 5) (C&D). The antibody titers were determined by indirect ELISA against UV-inactivated DENV1/DENV2 particles as indicated. (A, C) Total IgG titers against DENV1 or DENV2 as indicated on the top of the graphs. (B, D) Anti-DENV1 IgG1, IgG2a and IgG2b titers. Titers were determined as the lowest dilution factor that gives an OD value of 3 times the mean blank. Dotted line denotes the lowest dilution of the sera and a nominal titer of 50 or 66.7 was assigned to samples with titers below the lowest dilution.

Infection of adult female A129 mice via the intravenous (iv) route with 10^6^ PFU of DENV1 resulted in an asymptomatic infection with barely detectable viremia by plaque assay (S1A Fig). However, detection of anti-NS1 IgG antibodies in the sera of these mice 6 weeks post-infection (p.i.) confirmed that the virus had replicated productively (S1B Fig). High IgG antibody titers against homologous DENV1 and heterologous DENV2 virus were measured by ELISA in the sera from these DENV1-immune female mice (Fig 1A). The same assays were performed with the serum from 5–6 weeks old mice born to these DENV1-immune mothers. The total anti-DENV1 and anti-DENV2 IgG titers measured were 1 log lower than those measured in their mother (Fig 1C). Remarkably, the anti-DENV1 IgG antibody response was mainly (DENV1-immune mothers) or exclusively (mice born to the DENV1-immune mothers) of the IgG2a sub-class (Fig 1B & 1D).

**Fig 2:**
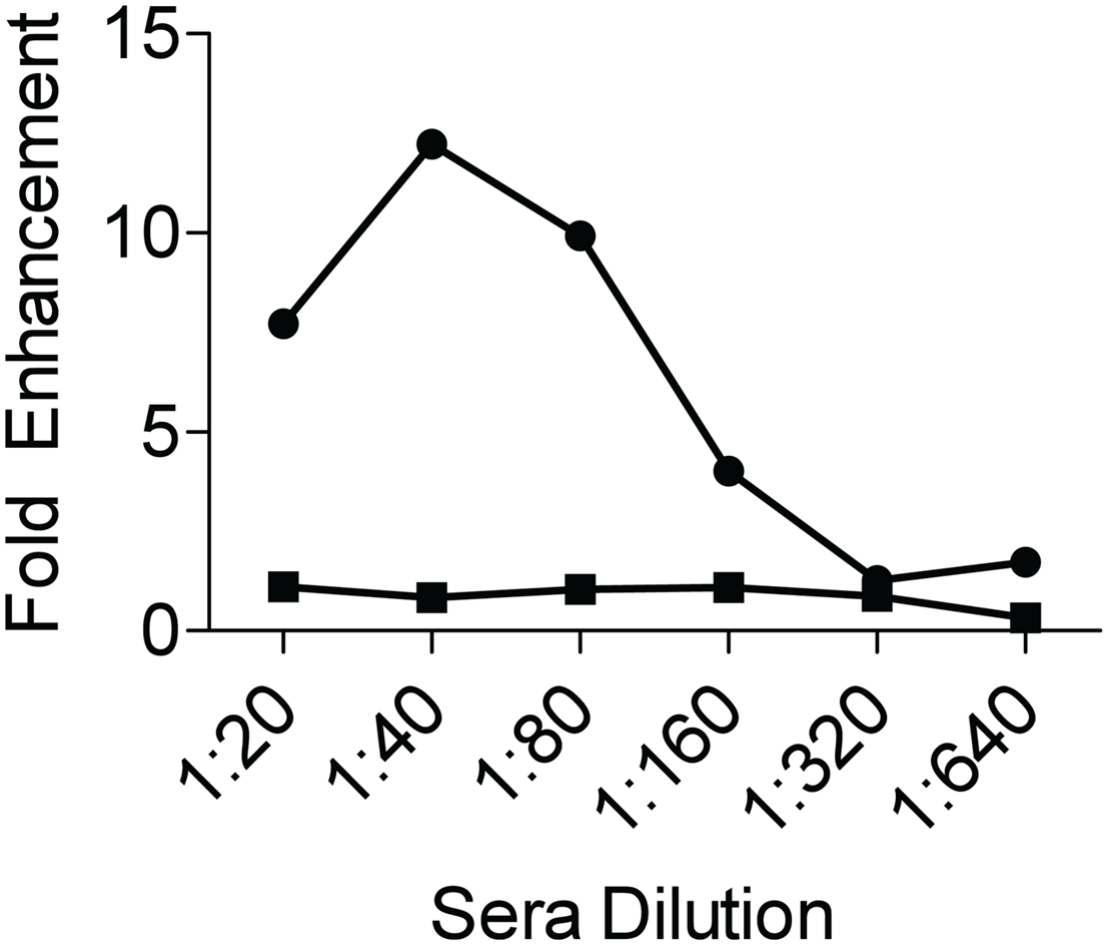
Enhancing properties of sera from 5–6 weeks old mice born to DENV1-immune mothers. K562 cells were infected with a mixture of DENV2-D2Y98P-PP1 and serially diluted pooled sera from mice born to DENV1-immune mothers (circle) or that from naïve mothers (square). Virus titers in supernatant were determined by plaque assay in BHK cells and expressed as fold enhancement (with respect to the average plaques obtained in the virus alone control).

Neutralizing capacity of the serum from DENV1-immune mothers and their offspring was also evaluated against DENV1 and DENV2 using classical *in vitro* plaque reduction neutralization test (PRNT). Results show that the sera from DENV1-immune mothers displayed some neutralizing capacity against both DENV1 and DENV2, with PRNT_50_ titers of about 140 and 50, respectively (Table 1). Expectedly, the neutralizing capacity of the sera from mice born to DENV1-immune mothers was significantly lower than that seen with their mother, with average PRNT_50_ values of 78 and 13 against DENV1 and DENV2, respectively. Finally, the enhancing properties against DENV2 of the sera from mice born to DENV1-immune mothers were assayed *in vitro*. Enhanced DENV2 infection of K562 cells was observed with serum dilutions ranging from 1/20 to 1/160 (Fig 2).

**Table 1:** Neutralizing properties of sera from DENV1-immune mothers and their offspring. Serially diluted (2-fold) heat-inactivated sera from DENV1-immune mothers (6 weeks p.i.), age-matched naïve mothers and 5–6 weeks old mice born to their respective mothers were mixed with either DENV1 or DENV2 prior to infection of BHK cells. The PRNT_50_ value was determined by nonlinear regression of the serum dilution factor that results in 50% reduction in the number of plaques as compared to the positive control (virus alone). Samples with PRNT_50_ values less than 1:10 were arbitrarily assigned a value of 5. Sera from DENV1-immune and naïve mothers were pooled whereas sera from mice born to their respective mothers were tested individually (n = 5) and are expressed as average ± SEM.

	DENV1	DENV2
Naïve mothers (Age-matched)	5.00	5.00
DENV1-immune mothers (6 weeks post-infection)	139.12	59.20
Mice born to naïve mothers	10.01 ± 3.02	6.33 ± 1.33
Mice born to DENV1 immune mothers	78.14 ± 10.69	13.30 ± 2.38

Together these *in vitro* data demonstrate that serum from mice born to DENV1-immune mothers cross-reacts with but does not neutralize effectively DENV2, which results in enhancement of infection.

### Enhanced disease severity upon DENV2 infection of A129 mice born to DENV1-immune mothers

**Fig 3:**
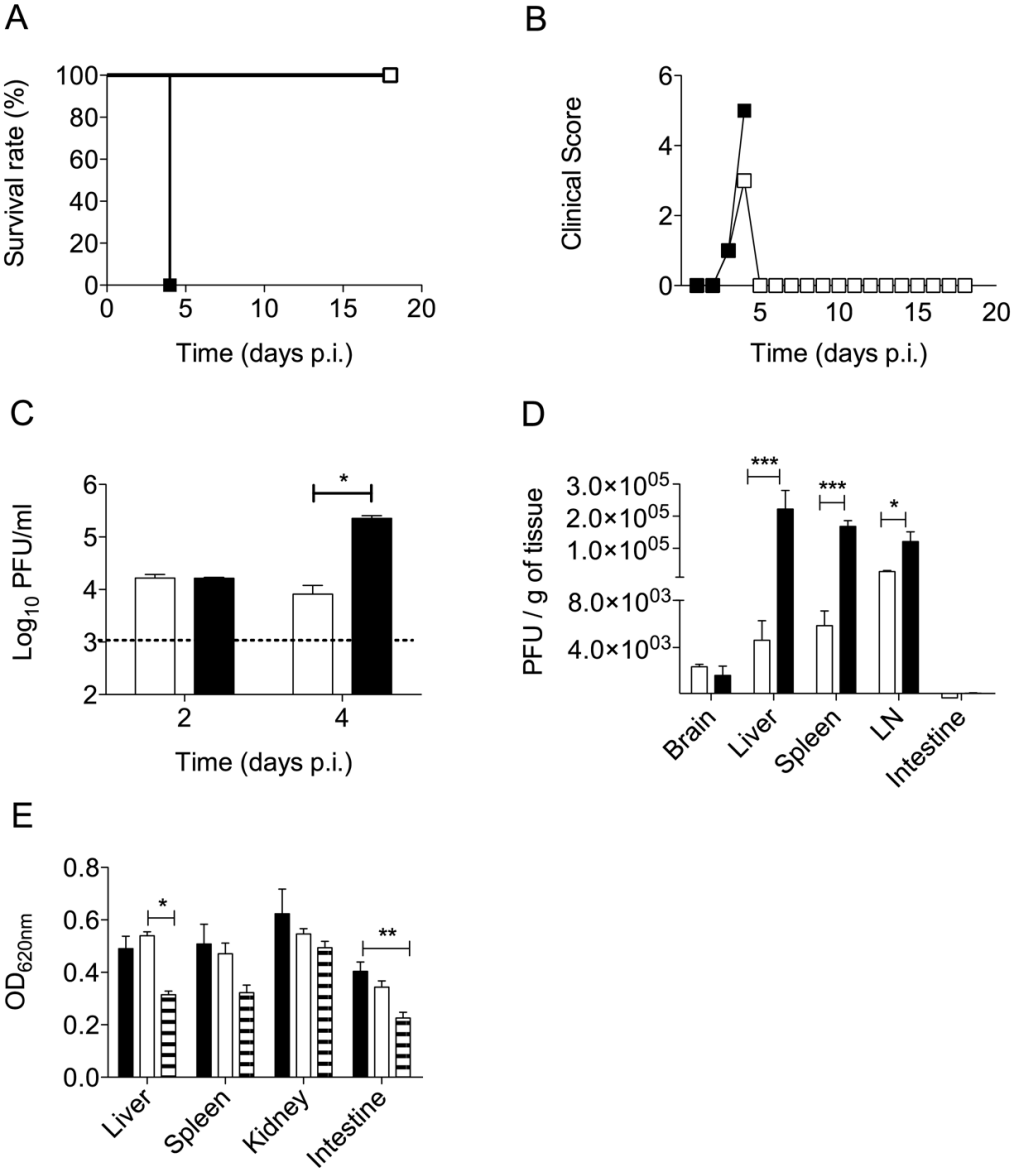
Survival rate, clinical score, viremia and organ viral titers in DENV2-infected mice born to DENV1-immune or naïve mothers. 5-6-weeks old A129 mice born to either DENV1-immune (black squares/bars) or naïve mothers (open square/bar) were iv infected with 10^6^ PFU of D2Y98P-PP1, a DENV2 strain. (A) Survival rate (n = 7–10). (B) Mean clinical score. 0: Healthy; 1: Ruffled Fur; 2: Hunched back; 3: Severe Diarrhea; 4: Lethargic; 5: Moribund. (C) Viremia. The number of infectious viral particles in serum was determined by plaque assay at day 2 and 4 p.i. (n = 5 mice per group per time point ± SEM). Dotted line denotes detection limit. (D) Viral load in brain, liver, spleen, brachial/ axillary lymph nodes, and intestines at day 4 p.i. was measured by plaque assay (n = 5 ± SEM). (E) Vascular leakage was assessed at day 4 p.i. using Evans’s blue dye. Five mice per group were iv injected with the dye and euthanized 2 hours later. After perfusion with PBS, various organs were harvested and processed for Evan’s blue dye extraction and quantification. Results are expressed as OD absorbance per gram of wet tissue (± SEM) and compared to uninfected control animals. Legend: DENV2-infected mice born to dengue naive mothers (open bar); DENV2-infected mice born to DENV1-immune mothers (black bar) and uninfected mice (striped bar). * *p*<0.05 based on 1-way ANOVA with Bonferroni’s post-test for 1C and 2-way ANOVA with Bonferroni’s post-test for 1D and E. For each panel, one representative set of at least three independent experiments is shown.

Five to six week old mice born to DENV1-immune females or naïve females were infected iv with 10^6^ PFU of DENV2 clinical isolate D2Y98P-PP1, which we have previously shown to trigger vascular leakage and death in AG129 mice upon primary infection [27, 28]. Here, A129 mice born to DENV1-immune mothers consistently and uniformly died by day 4 post-DENV2 infection (p.i.) whereas the infected age-matched A129 mice born to naïve mothers survived throughout the experimental period (Fig 3A). Both groups started to display clinical symptoms including hunched back and severe diarrhea at the end of day 3 and at day 4 p.i. However, while the mice born to naïve mothers recovered by day 5 p.i., mice born to DENV1-immune mothers rapidly became lethargic and moribund at which stage they were euthanized (Fig 3B). None of the infected mice displayed signs of paralysis or central nervous system (CNS) involvement.

Comparable viremia titers were measured at day 2 p.i. in both animal groups (Fig 3C). However, viremia was 1 log higher at day 4 p.i. in mice born to DENV1-immune mothers compared to the age-matched control mice (Fig 3C). The viral loads in perfused liver, spleen and brachial/axillary lymph nodes were also significantly higher at day 4 p.i. in mice born to DENV1-immune mothers (Fig 3D). Negligible levels of virus were detected in the brain from the infected mice. Furthermore, as increased vascular leakage was the main feature in the maternal ADE model in AG129 mice [31], we also assessed the vascular permeability in A129 mice. Interestingly, moderate (less than 2-fold increase compared to uninfected controls) and comparable vascular leakage was measured in both groups of DENV2-infected A129 mice born to naïve or DENV1-immune mothers (Fig 3E).

Together, these data indicate that DENV2 infection of young A129 mice born to DENV1-immune mothers led to increased disease severity characterized by lethality and increased viral loads in the blood and several organs. However, these mice did not experience further increase in vascular leakage as compared to their counterparts born to naïve mothers.

### Severe liver damage and elevated transaminase levels in DENV2-infected mice born to DENV1-immune mothers

**Fig 4:**
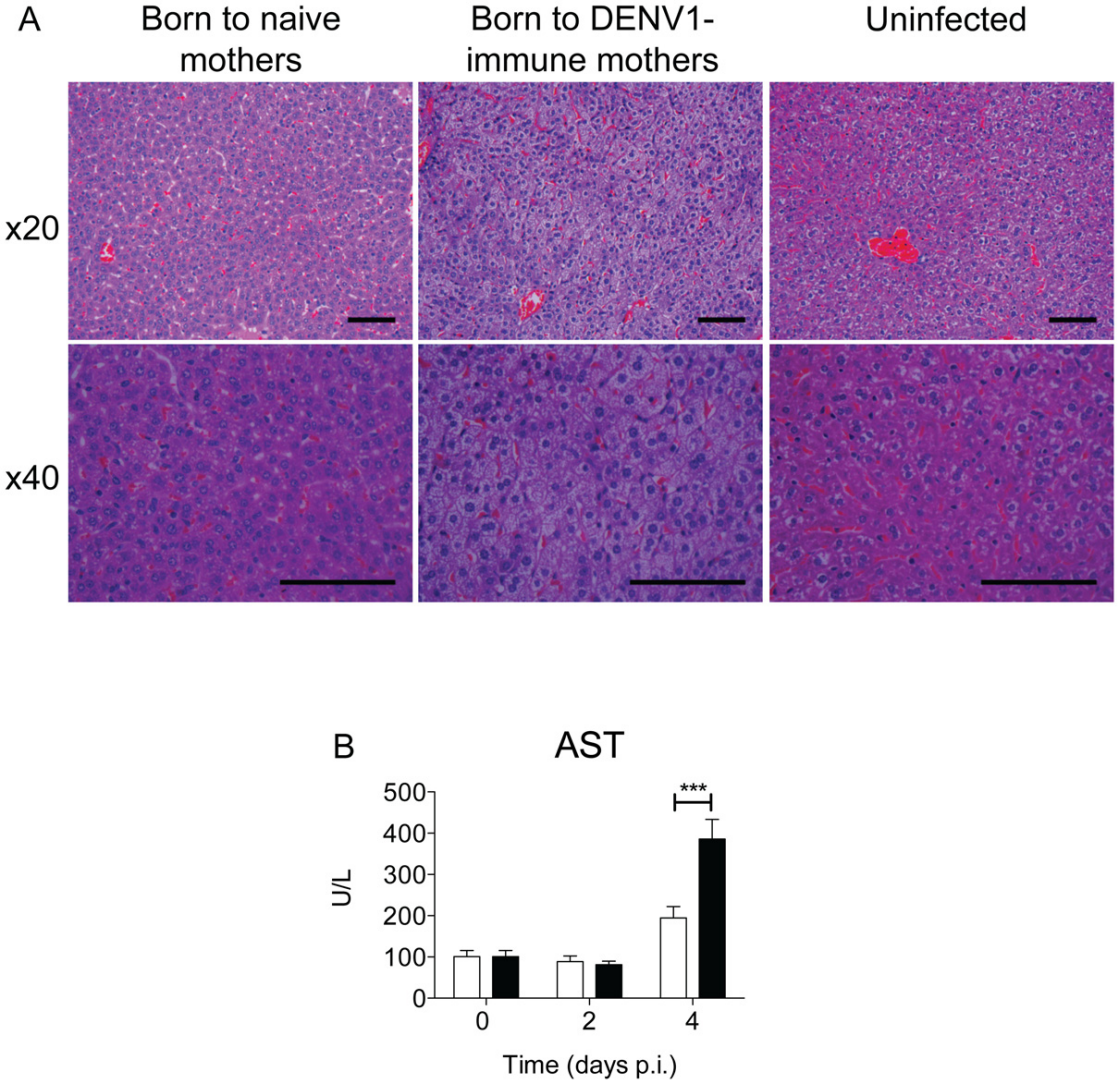
Liver histology and function in DENV2-infected mice born to DENV1-immune. 5-6-weeks old A129 mice born to either DENV1-immune (black bars) or naïve mothers (open bars) were iv infected with 10^6^ PFU of D2Y98P-PP1. (A) Histological analysis of the liver at day 4 p.i. of DENV2-infected mice born to dengue naïve or DENV1-immune mothers and uninfected mice as indicated (n = 3). Images were taken at 20x and 40x magnification. Representative sections from two independent experiments are shown (scale bar– 100μm). (B) Serum levels of aspartate transaminase (AST) were determined at the indicated time points (n = 5 ± SEM per time point), * *p*<0.05 based on 1-way ANOVA with Bonferroni’s post-test.

To gain further insights into the pathology displayed by the DENV2-infected mice born to DENV1-immune mothers, histology analysis was conducted. Upon H&E staining, severe liver damage was observed at day 4 p.i. in mice born to DENV1-immune mothers, characterized by widespread cytoplasmic vacuolation of the hepatocytes (Fig 4A). These mice displayed healthy hepatocytes at the earlier time point of day 2 p.i. (S2 Fig). Liver damage observed at day 4 p.i. correlated with elevated systemic levels of aspartate transaminase (AST) (Fig 4B). Instead, mice born to dengue naïve mothers displayed healthy liver (Fig 4A) with normal AST levels (Fig 4B). In addition, no evident damage was observed in the spleen and intestine from DENV2-infected mice born to naïve and DENV1-immune mothers at day 4 p.i. (S2 Fig), thus suggesting that maternal antibody-mediated disease enhancement targets mainly the liver.

**Fig 5:**
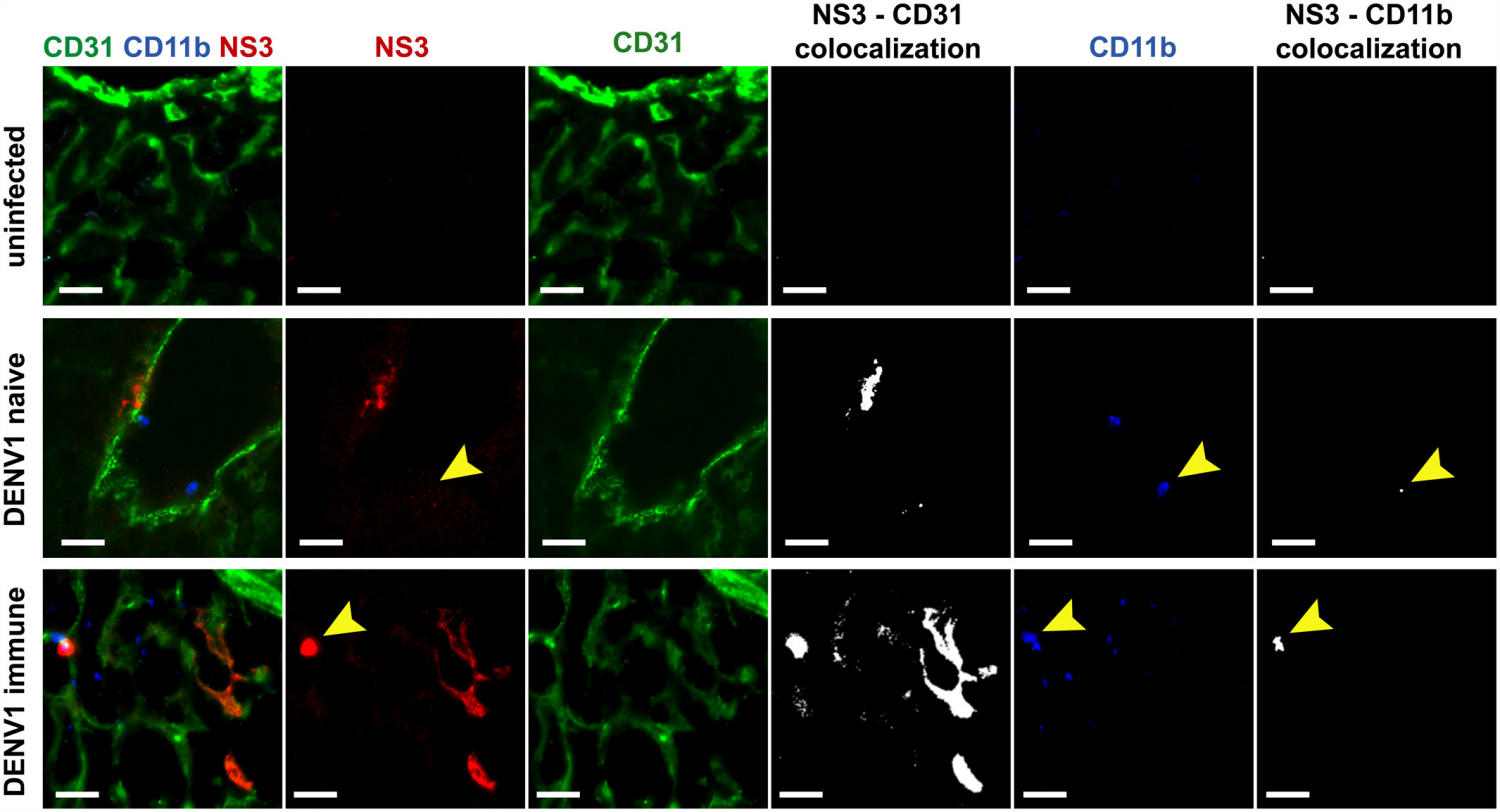
DENV antigen detection in the liver from DENV- infected A129 mice born to DENV1-immune or naïve mothers. Five to six weeks old A129 mice born to either DENV1-immune or naïve mothers were iv infected with 10^6^ PFU of D2Y98P-PP1. At day 4 p.i., tissues were harvested, frozen-sectioned, and stained for DENV NS3 (red) and cellular markers against endothelial cells (CD31, green), and monocytes (cd11b, blue). Representative sections from 2 independent experiments, each with n = 3 animals are shown (scale bar– 10μm). Individual channels corresponding to the merged images and co-localization images are presented.

A previous study reported the accumulation of DENV particles in liver sinusoidal endothelial cells in an ADE model where AG129 mice were administered enhancing antibodies prior to DENV2 infection [30]. Similarly, we found that DENV NS3 protein mainly co-localized with CD31^+^ cells, a marker for endothelial cells and to a lesser-extent with CD11b^+^ cells, a marker primarily expressed on monocytes/macrophages in mice born to DENV1-immune mothers (Fig 5). In mice born to dengue naïve mothers, NS3 staining showed co-localization with similar cell markers, although overall the levels of infection appeared reduced (Fig 5). These observations thus indicate that hepatocytes are not the main target cell population for DENV in both infected groups. This therefore suggests that the extensive cytoplasmic vacuolation of hepatocytes seen in DENV2-infected mice born to DENV1-immune mothers is not due to a direct cytolytic viral effect and correlates with the possible heightened presence of the virus inside the liver sinusoidal endothelial cells.

### DENV2-infected mice born to DENV1-immune or naïve mothers display comparable changes in their blood parameters

Blood parameters were assessed at day 2 and 4 p.i in both infected groups. Compared to uninfected mice, both DENV2-infected groups displayed increased neutrophils and reduced lymphocytes and monocytes counts, which was more evident at day 4 p.i. (S3 Fig). Thrombocytopenia was also seen for both infected animal groups as evidenced by the lower platelet counts measured at both day 2 and 4 p.i. Instead, there was no significant difference in the hematocrit between infected groups and uninfected control, reflecting the moderate vascular leakage measured in the infected mice (Fig 3). The only significant difference between mice born to DENV1-immune mothers and mice born to naïve mothers was a lower lymphocyte count at day 4 p.i. in the former group. Therefore, it appears that while DENV2 infection of A129 mice affects a number of blood parameters such as white blood cells, neutrophils, lymphocytes, monocytes, and platelets counts, these differences are unlikely to explain the enhanced disease severity seen in mice born to DENV1-immune mothers since they are also observed in mice born to dengue naïve mothers.

### Increased levels of pro-inflammatory cytokines and chemokines in DENV2-infected mice born to DENV1-immune mothers

**Fig 6:**
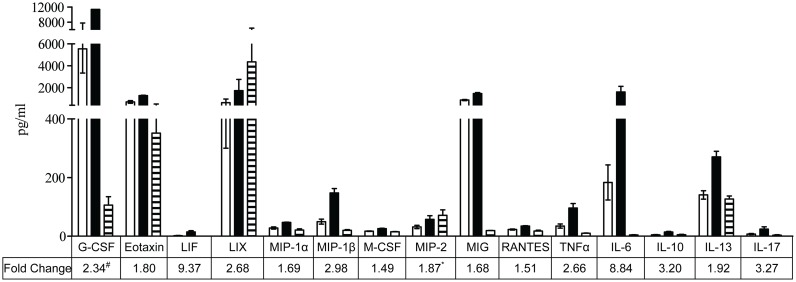
Systemic levels of differentially modulated cytokines and chemokines in DENV2-infected A129 mice born to DENV1-immune or naïve mothers at day 4 p.i. Five to six weeks old A129 mice born to either DENV1-immune or naïve mothers were iv infected with 10^6^ PFU of D2Y98P-PP1. At day 2 or 4 p.i., the mice were terminally bled and the systemic levels of cytokines were determined by multiplex ELISA. Average levels of the differentially modulated cytokines (n = 5 each for 2–3 independent experiments) in the sera of DENV2-infected mice born to DENV1-immune mothers (black bars), DENV2-infected mice born to naïve mothers (open bars) and age-matched uninfected mice (stripe bars) were shown and error bar denotes standard deviation between experiments. Fold change were obtained by taking the average cytokine levels of the DENV2-infected mice born to the naïve mothers as reference. Legend: ^#^ Fold change is estimated as levels in DENV2-infected mice born to DENV1-immune mothers were above upper detection limit of the multiplex assay. *Levels in DENV2-infected mice born to DENV1-immune mothers were not significantly different from the basal levels in the uninfected control mice.

A hallmark of DHF/DSS is the heightened expression of cytokines that results in various pathological complications [3, 34]. To gain further insights into this A129 ADE model and identify the soluble mediators that could contribute to disease enhancement, the levels of 32 different cytokines and chemokines were measured in the sera from DENV2-infected mice born to naïve or DENV1-immune mothers. At the early infection time point of day 2 p.i., only two cytokines, namely Granulocyte-colony stimulating factor (G-CSF) and monokine induced by IFN-γ (MIG), were significantly reduced in DENV2-infected mice born to DENV1-immune mothers compared to DENV2-infected mice born to naïve mothers (average values of 2026.72 pg/ml vs 7080.75pg/ml for G-CSF and 128.75pg/ml vs 395.42pg/ml for MIG) at day 4 p.i. when clinical symptoms were observed, a greater number of cytokines became differentially expressed. Higher systemic levels of G-CSF, Eotaxin, LIF, LIX, MIP-1a, MIP-1β, MCP, MIP-2, MIG, RANTES, TNFα, IL-6, IL-10, IL-13 and IL-17 were measured in mice born to DENV1-immune mothers compared to DENV2-infected mice born to naïve mothers (Fig 6). When expressed as fold change, these expression levels range from 1.5 (M-CSF) to 9.37 (LIF) fold-increase (Fig 6). These increased levels of pro-inflammatory cytokines and chemokines measured in DENV2-infected mice born to DENV1-immune mothers therefore support that these mice experienced an exacerbated inflammatory response.

### Key role of TNFα in disease severity

**Fig 7:**
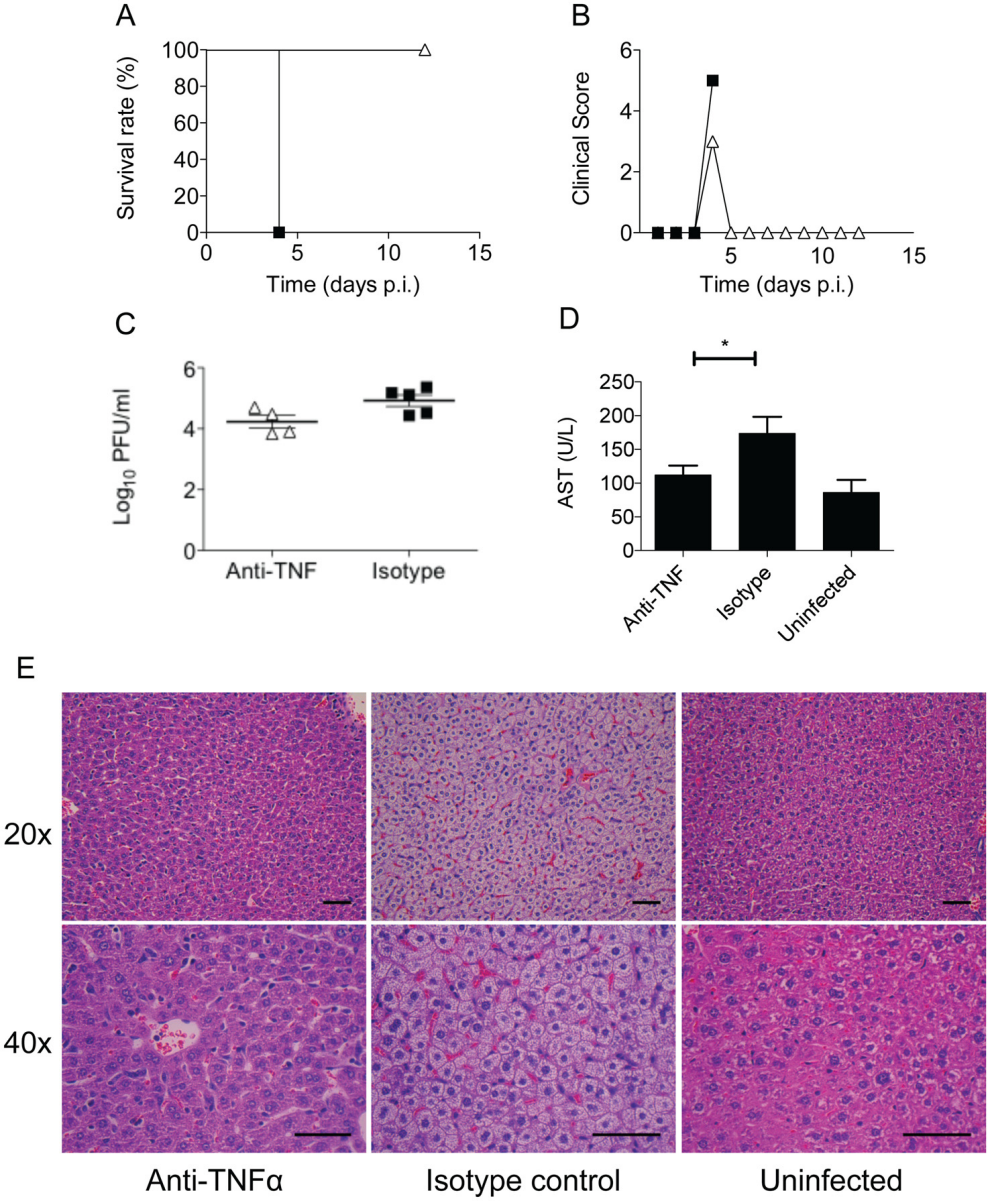
Anti-TNFα treatment of DENV2-infected mice born to DENV1-immune mothers. Five to six-weeks old A129 mice born to DENV1-immune mothers were infected iv with 10^6^ PFU of D2Y98P-PP1. At day 2 p.i., the mice were injected iv with 100 μg of anti-TNFα neutralizing antibody (open square) or 100μg of isotype control antibody (black square). (A) Survival rate and (B) mean clinical score of anti-TNFα-treated and isotype control antibody-treated DENV2-infected mice born to DENV1-immune mothers (n = 7–8). (C) Viremia and (D) serum AST levels of anti-TNFα-treated and isotype control at day 4 p.i. (n = 5 ± SEM, * *p* < 0.05 based on Mann-Whitney test with respect to isotype control). (E) Histological analysis of the liver of anti-TNFα-treated mice, isotype antibody-treated mice harvested at day 4 p.i., and uninfected mice as indicated (n = 3 per group). Images were captured at 20x and 40x magnification. Representative sections from two independent experiments are shown (scale bar– 100μm).

Patients with severe dengue have been reported to display elevated levels of TNFα [35] and in several AG129 ADE models, TNFα blocking antibodies were found to delay the death of DENV2-infected mice [30, 31]. Consistently, increased TNFα levels were measured in DENV2-infected A129 mice born to DENV1-immune mothers at moribund stage (day 4 p.i.) (Fig 6). To assess the role of TNF-α in this A129 ADE model, DENV2-infected mice born to DENV1-immune mothers were administered with a TNF-α blocking antibody or an isotype control at day 2 p.i. Survival and clinical symptoms were monitored and indicated that the anti-TNFα-treated mice survived throughout the experiment while the isotype control mice were moribund by day 4 p.i. (Fig 7A & 7B). Comparable viremia titers were measured at day 4 p.i. in both anti-TNFα and isotype control treated groups (Fig 7C), thus indicating that the absence of disease enhancement in the anti-TNFα treated group was not due to reduction in virus loads. In contrast, AST level was significantly lower in the anti-TNFα-treated group compared to the isotype control group (Fig 7D), suggesting that the mice had reduced liver damage. This was confirmed by histology analysis whereby mice treated with anti-TNFα antibody displayed no obvious liver damage. In contrast, isotype control mice showed substantial cytoplasmic vacuolation (Fig 7E). Together, these data support that liver damage is the likely cause of death in this ADE model and indicate that TNFα plays a key role in this process.

### Role of IL-6 and IFNγ in disease severity

**Fig 8:**
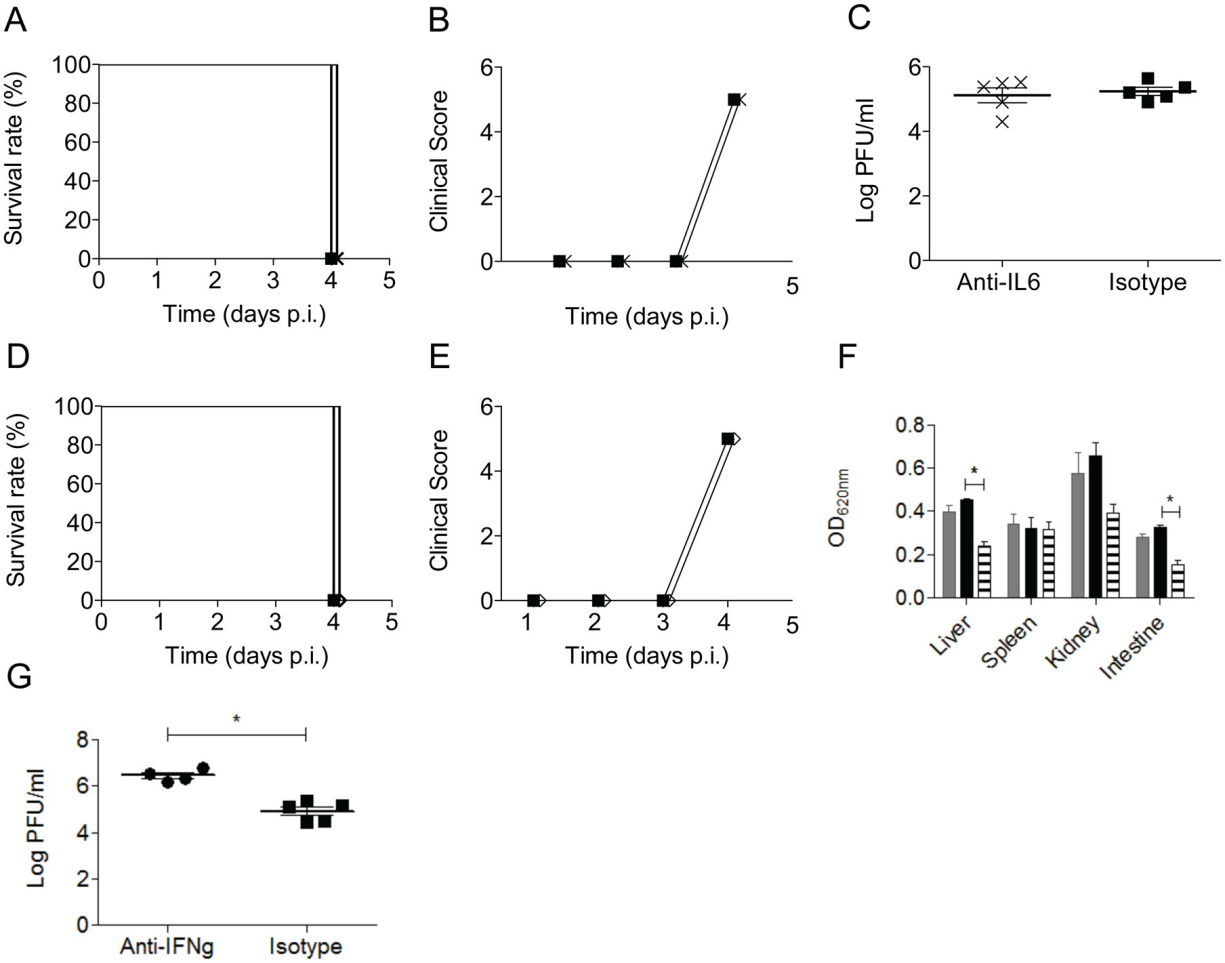
Anti-IL6 and anti-IFNγ treatment of DENV2-infected mice born to DENV1-immune mothers. Five to six weeks old A129 mice born to DENV1-immune mothers were infected iv with 10^6^ PFU of D2Y98P-PP1. At day 2 p.i., the mice were injected iv with 100μg of anti-IL6 or anti-IFNγ neutralizing antibody, or 100μg of isotype control antibody. (A) Survival rate and (B) mean clinical score of anti-IL6-treated (cross) and isotype control antibody-treated (black square) DENV2-infected mice born to DENV1-immune mothers (n = 7–8). (C) Viremia (n = 5 ± SEM) of anti-IL6-treated and isotype control antibody-treated DENV2-infected mice born to DENV1-immune mothers (*p* = 0.111 based on Mann-Whitney test). (D) Survival rate and (E) mean clinical score of anti-IFNγ-treated (open diamond) and isotype control antibody-treated (black square) DENV2-infected mice born to DENV1-immune mothers (n = 7–8 per groups). (F) Vascular leakage (n = 3–4 ± SEM) of anti-IFNγ-treated (grey bars), isotype control antibody-treated (black bar) DENV2-infected mice born to DENV1-immune mothers and uninfected mice (stripe bar) (* *p* < 0.05 based on Kruskal-Wallis test with Dunn’s post test). (G) Viremia (n = 4–5 ± SEM) of anti-IFNγ-treated and isotype control antibody-treated DENV2-infected mice born to DENV1-immune mothers (* *p* < 0.05 based on Mann-Whitney test).

Among the panel of cytokines and chemokines measured, the levels of IL-6 were highly elevated in the DENV2-infected mice born to DENV1-immune mothers compared to mice born to naïve mothers (Fig 6). In addition, elevated levels of this cytokine have been reported in severe dengue patients [36]. In order to investigate the role of IL-6 in this A129 ADE model, DENV2-infected mice born to DENV1-immune mothers were treated with an anti-IL6 blocking antibody at day 2 p.i. In sharp contrast to what was observed when TNFα was neutralized at the same time point, no differences were observed in terms of survival, clinical scores or viremia in the anti-IL6 treated mice compared to the isotype control group (Fig 8A–8C). This thus suggests that increased IL-6 production in mice born to DENV2-immune mothers is not a major contributor to the enhancement of disease severity.

Furthermore, the role of IFNγ in this ADE model was evaluated using a similar strategy. We were particularly interested to test whether the presence of an intact IFNγ signaling pathway in A129 mice plays a role in the limited vascular leakage seen in this ADE model, compared to the ADE model in AG129 mice for which we previously reported extensive vascular permeability as the primary cause of morbidity for the infected mice that have acquired maternally transferred antibodies [31]. Hence, DENV2-infected A129 mice born to DENV1-immune mothers were treated with a neutralizing antibody against IFN-γ at day 2 p.i. There were no significant differences in survival, clinical scores or extent of vascular leakage between the anti-IFNγ-treated mice and the isotype control mice (Fig 8D–8F). However, increased viremia was measured in the anti-IFNγ-treated animals compared to the group treated with the isotype control antibody (Fig 8G). Together, these data support that IFNγ helps limit viral replication in this ADE model but does not seem to play a critical role in the extent of vascular leakage detected in various organs.

## Discussion

ADE mouse models involving the passive administration of enhancing antibodies (polyclonal immune serum or purified monoclonal antibodies) prior to DENV infection have been widely reported by several groups [29, 30, 37]. This approach is straight forward and allows controlling the dose and nature of the antibodies that are administered. In comparison, the maternal ADE model may be seen as less “artificial” since it closely mimics a situation that is encountered in humans. Since heterologous secondary infection in mice does not lead to enhanced disease severity [38], the maternal ADE model represents the second best option to mimic a secondary heterologous infection. Furthermore, it allows the investigation of specific pathogenesis aspects that cannot be addressed in the passive administration ADE model, such as the role of breast milk antibodies in disease enhancement for example. With the recent advances in dengue vaccine development, the protective or enhancing properties of vaccination-induced maternal antibodies in infants, or the possible interference of maternal antibodies on vaccination efficacy in babies and young children are interesting topics that our maternal ADE model can also help investigate.

The vast majority of studies reported so far have used the immunocompromised mice AG129 mice, which are deficient in type I and type II IFN receptors. AG129 mice allow productive infection upon infection with various DENV strains that may be accompanied by relevant clinical manifestations. However, the lack of an intact and functional type II IFN signaling pathway in these mice precludes or limits the investigation of a number of aspects in particular the host adaptive immune responses to DENV infection. A couple of very recent studies have reported the use of mice in which absence of type I IFN receptor is restricted to myeloid cell subsets (dendritic cells and macrophages) [39, 40]. In this model, mice succumbed to DENV infection thereby offering a novel *in vivo* platform for pathogenesis studies and therapeutic testing [39, 40].

Here, we have used A129 mice, which are deficient in type I IFN receptor but have an intact and functional type II IFN pathway. Infection of adult A129 female mice with a high dose of DENV1 via the iv route resulted in an asymptomatic barely detectable viremia followed by the production of anti-DENV1 antibody titers including antibodies against both structural and non-structural (NS1) proteins, thus indicating DENV1 productive replication. We then showed that 5-week old A129 mice born to these DENV1-immune mothers, succumbed to DENV2 infection within 4 days whereas age-matched mice born to dengue naïve mothers survived the infection. Increased virus titers in the blood and several organs, and severe liver damage were observed in the moribund animals.

In a recently published work using the AG129 mouse strain, we reported that 5-week old mice born to DENV1-immune mothers displayed disease severity enhancement characterized by earlier death, increased virus titers and extensive vascular leakage compared to age-matched mice born to dengue naïve mothers [31].

Whereas disease enhancement is clearly observed for both mouse models, it is interesting to note that the clinical manifestations differ. Indeed, in the AG129 mouse model extensive vascular leakage was observed with 8–18 fold increases in OD values in mice born to DENV1-immune mothers compared to uninfected controls [31]. However, no significant liver damage was seen in the infected mice [31]; on the other hand enhancement of disease severity in the A129 ADE model correlated with severe liver damage but moderate vascular leakage. Given that AG129 and A129 differ by the presence of a functional type II IFN pathway, one could hypothesize that IFNγ helps limit vascular leakage in A129 mice. However, neutralization of IFNγ with a blocking antibody did not impact the extent of vascular leakage measured in the A129 ADE mouse model. This thus supports a negligible role for IFNγ in vascular leakage observed in the A129 ADE mouse model. Instead, we showed that IFNγ clearly limits viral replication, as previously reported in an AG129 model [41]. The fact that the increased viral titers did not translate into earlier death of the animals may be explained by the acute nature of our model where mice die within 4 days.

When comparing the AG129 and A129 ADE models, we propose that the differential route of DENV2 infection as well as the different infectious doses may impact the disease progression and manifestations. While in AG129 10^3^ PFU of DENV2 were administered subcutaneously (sc), A129 were infected via the iv route with 10^6^ PFU, due to their known greater resistance to dengue infection [37, 42]. Upon sc infection, dendritic cells are likely to be the first host cells infected by DENV [42, 43], which will then migrate to the draining lymph nodes where naïve T cells will be activated followed by systemic dissemination of the virus and interaction with maternally acquired heterotypic enhancing antibodies. Instead, iv administration of DENV2 allows immediate interaction of the virus with maternal enhancing antibodies followed by uptake into FcR-bearing cells (mainly monocytes), thus bypassing or minimizing interactions with and infection of DCs. Thus it is possible that in A129 mice, activation of the adaptive immunity through DCs is minimal and leads to acute death (day 4 p.i.) of the infected mice even before further increase in vascular leakage can be detected. Furthermore, the direct delivery of high amounts of virus particles into the bloodstream is likely to overwhelm the highly vascularized organs such as spleen and liver where the greatest increase in virus titers was measured compared to mice born to naïve dams, leading to organ damage and failure.

Nevertheless, the relevance of liver involvement in dengue disease is supported by several case report studies where dengue infection has been associated with involvement of multiple organs, one of the most common being the liver [44–46]. The range of involvement includes asymptomatic elevation of liver aminotransferases to severe manifestations in form of acute liver failure [44, 47–49]. Other mouse models of dengue infection have also reported hepatic damage characterized by cellular infiltration and vacuolation of hepatocytes, accompanied by transient increase in the ASL and ALT levels and in some cases substantial vascular leakage [25, 27, 50–56].

Furthermore, Zellweger and colleagues previously showed enhanced infection of liver sinusoidal endothelial cells in AG129 mice upon passive transfer of enhancing anti-dengue antibodies [30]. In our A129 ADE model, we also observed enhanced DENV infection of liver endothelial cells in mice born to DENV1-immune mothers compared to mice born to naïve mothers. These observations thus suggest that the extensive cytoplasmic vacuolation of hepatocytes observed in these mice is not a consequence of a direct cytopathic viral effect. A study reported that anti-NS1 antibodies in absence of dengue infection can result in liver damage [57]. The pathological role of maternal anti-NS1 antibodies in our ADE model remains to be investigated. Alternatively hepatocytes apoptosis may result from deregulated cytokines production, in particular TNFα. However, in a recent study investigating liver damage in 13 autopsy cases of DHF/DSS, DENV proteins were detected in hepatocytes and Kupffer cells but not in endothelial cells [58].

Among the cytokines that were up-regulated in the DENV2-infected mice born to DENV1-immune mothers, TNFα was identified as a critical player as its *in vivo* neutralization at day 2 p.i. fully protected the animals from disease enhancement. Importance of TNFα in dengue disease severity has been illustrated in various mouse models. In primary DENV2 infection models using either immunocompetent [59] or AG129 [25] mice, blocking TNFα using an antibody partially protected the animals from liver damage and vascular leakage, respectively. DENV2 infection of TNFα^-/-^ mice resulted in significant reduction of hemorrhage compared to wild-type infected mice [18]. Such observations are not restricted to DENV2 infection. Indeed, complete protection was obtained in a DENV3 lethal AG129 model upon treatment with anti-TNFα antibodies [60]. Furthermore, the pathological role of TNFα has been substantiated in other *in vivo* ADE models where anti-TNFα treatment of infected mice improved their mean survival time [30, 31]. Hence, it is clear that TNFα is a key pathogenic cytokine responsible for the different disease manifestations such as liver damage and vascular leakage in various murine dengue models. However, the role of TNFα in human dengue is less clear. While several studies have identified a positive correlation of increased TNFα levels with dengue disease severity [49, 61], others have noticed no significant differences in expression levels of this cytokine during severe or mild dengue [62, 63]. Inconsistencies in TNFα levels in these cytokine profiling studies of dengue patients have largely been attributed to possible differences in collection time points and discrepancies in the assessment of disease severities [64]. Evaluation of genetic predisposition to severe dengue also yields conflicting conclusions with regards to TNFα polymorphisms. High producing TNFα allele 308A has been determined to be a risk factor for bleeding manifestations during severe dengue [65, 66]. However, the same allele has also been identified to be protective against severe dengue [67]. A dual role for TNFα may be proposed which could act as both a protective and detrimental cytokine, depending on the stage of infection. This hypothesis is supported by an *in vitro* study showing that early post-infection, TNFα functions primarily as a pro-survival signal and activates NF-κB [68]. As the infection progresses however, the infected cells become less sensitive to TNFα-mediated NF-κB stimulation and undergo apoptosis.

In conclusion, we report here a novel mouse model of disease enhancement that is mediated by maternally acquired antibodies in the A129 background. It recapitulates a number of clinical manifestations observed in severe dengue patients including increased vascular permeability, thrombocytopenia, lymphopenia, cytokine storm and liver pathology. Since A129 mice are less immunocompromised than AG129, this model offers a better platform not only to study dengue pathogenesis, but also to evaluate the efficacy of therapeutic and vaccine candidates. Specifically, this liver disease-associated model may indeed prove useful for testing the efficacy of novel dengue therapeutics in alleviating hepatic manifestations during severe dengue. In addition, with recent advances in dengue vaccines clinical development, this maternal ADE model may help investigate the impact of maternal antibodies on vaccination efficacy in infants and provide vaccination strategy guidelines.

## Materials and Methods

### Ethics statement

All the animal experiments were carried out under the guidelines of the National Advisory Committee for Laboratory Animal Research (NACLAR) in the AAALAC-accredited NUS animal facilities (http://nus.edu.sg/iacuc/). NUS has obtained a license (#VR008) from the governing body Agri-Food & Veterinary Authority of Singapore (AVA) to operate an Animal Research Facility. The animal experiments described in this work were approved by the IACUC from NUS under protocol number 2013–04751.

### Virus strains and growth conditions

The DENV2 D2Y98P-PP1 strain was derived from a 2000 Singapore clinical isolate (Genbank accession number #JF327392) [69]. DENV1 [Dengue 1 05K3903DK1 (Genbank accession number #EU081242)] was isolated from a patient during a DEN outbreak in Singapore in 2005 [70]. The *Aedes albopictus* C6/36 cell line (American Type Culture Collection [ATCC #CRL-1660]) was used for propagation of all the DENV strains as described previously [27]. C6/36 cells were maintained in Leibovitz’s L-15 medium (GIBCO) supplemented with 10% fetal calf serum (FCS). Virus stocks were stored at -80°C.

#### Measurement of DENV specific total IgG, IgG isotypes and anti-NS1 antibodies

The levels of systemic IgG antibodies against DENV1 or DENV2 were determined by indirect enzyme-linked immunosorbent assay (ELISA). Briefly, 96-well plates (Corning costar, NY, USA) were coated overnight at 4°C with 150 ng/well of UV-inactivated DENV1 or DENV2 or 10 ng/well of purified NS1 protein [70] in PBS. Serially-diluted serum samples were added to the wells and incubated for 1 h at 37°C. HRP-conjugated goat anti-mouse IgG (H+L) (Bio-rad) at 1:3000, or anti-mouse IgG1, IgG2a and IgG2b (Abcam) secondary antibody was used at a 1:10000 dilution. Detection was performed using O-phenylenediamine dihydrochloride substrate SigmaFast (Sigma Aldrich) according to the manufacturer’s instructions. The reaction was stopped upon adding 50 μl of 1M sulphuric acid and absorbance was read at 490nm using an ELISA plate reader (Bio-rad model 680). The absorbance values against log dilution factors were then plotted and respective titres were determined by taking the absorbance cutoff at three times the mean background.

#### *In vitro* ADE assay

Pooled sera from 5–6 weeks old mice born to naïve or DENV1-immune mothers were heat-inactivated at 56°C for 30 minutes. Serially diluted mice sera (2-fold dilutions, starting at 1/20 dilution) were incubated with 10^3^ PFU of DENV2 (D2Y98P-PP1) for 1 h at 37°C. K562 cells were then infected with the suspensions for 2 h at 37°C at a multiplicity of infection (MOI) of 0.01 before transferring the infected cells in 48-wells flat bottom tissue culture plates (Falcon) at 37°C, 5% CO_2_. Virus titers in the culture supernatant after two days of incubation were determined by plaque assay in BHK cells. Fold enhancement for each dilution was calculated by normalizing the titers at respective sera dilution with the average titers in K562 cells infected with the virus in the absence of serum.

#### *In vitro* plaque reducing neutralizing assay (PRNT)

Serial dilutions (2-fold, starting 1/10 dilution) of heat-inactivated serum from mice born to DENV1-immune or naive mothers was first prepared with RPMI 1640, 2% FCS (Life Technologies), containing 500 PFU/ml of virus. The suspensions were then incubated at 37°C for 1 h. A positive control with virus alone was also included. Plaque assay was then carried out in BHK-21 cells (in triplicates) as described above. The percentage of neutralization was determined by comparing the number of plaques obtained with each serum dilution to that obtained with the positive control. PRNT_50_ was determined by nonlinear regression as the serum dilution factor for which 50% reduction in the number of plaques (with respect to the virus control) was obtained. Samples with PRNT_50_ values less than 1:10 were arbitrarily assigned a value of 5.

### Plaque assay

Plaque assay was carried out in BHK-21 cells as described previously [27]. Briefly, 1x10^5^ cells BHK-21 were seeded in 24- well plates (NUNC, NY, USA). BHK-21 monolayers were infected with 10-fold serially diluted viral suspensions or mice sera. After 1h incubation at 37°C and 5% CO_2_, 1% (w/v) carboxymethyl cellulose was added to the wells. After incubating 4 days for D2Y98P-PP1 or 5 days for DENV1, the cells were fixed with 4% paraformaldehyde and stained with 1% crystal violet for a minimum of 30 min. Then the plates were thoroughly rinsed with water, before counting the plaques. Results were expressed as log_10_ [mean ± SD] of plaque forming units (PFU) / ml of serum measured in 5 mice per group per time point. The limit of detection for the assay was set at 10^2^ PFU per ml.

### ADE infection mouse model

A129 mice (129/Sv deficient in IFNα/β receptor) breeders were obtained from B&K Universal (UK). They were housed and bred under specific pathogen-free conditions in individual ventilated cages. Five to six-week old A129 female mice were infected with 10^6^ PFU of DENV1 per mouse intravenously (iv), which led to asymptomatic transient viremia. One week post-infection, after virus clearance, the females were mated with naive 6-week old A129 males and the offspring were weaned at 21 days of age. Uninfected A129 females were used to give birth to naive controls. At 5–6 weeks of age, mice born to DENV1-immune or naive mothers were administered with 10^6^ PFU of DENV2 (D2Y98P-PP1) via the iv route (0.1 ml in sterile PBS). The infected animals were monitored daily for clinical symptoms. The scoring system used was: 0- Healthy; 1- Ruffled Fur; 2- Hunched back; 3- Severe Diarrhea; 4- Lethargic; 5- Moribund. Survival rate was derived from the number of mice that were euthanized at moribund stage as evidenced by severe diarrhea and extreme lethargy as described previously [27, 28].

### Organ processing for viral titer determination

Infected mice were euthanized and perfused extensively with sterile PBS. Pooled left and right brachial and axillary lymph nodes, brain, intestine, spleen, and liver were harvested and homogenized using a mechanical homogenizer (Omni) for 5 minutes in 1 ml RPMI 1640 at medium speed on ice. Thoroughly homogenized tissues were centrifuged at 14,000 rpm for 10 min at 4°C to pellet debris and then the supernatant was filtered using a 0.22 μm diameter pore size filter. The level of infectious virus within the filtrate is thus considered representative of the total level of infectious virus present in the harvested organ. Ten-fold serial dilutions of each tissue homogenate (from neat to 1: 10^4^) were assayed in a standard virus plaque assay on BHK-21 cells as described above. Five mice per time point per group were assessed.

### Histology

Mice were euthanized and tissues (intestines, spleen and liver) were harvested and immediately fixed in 4% paraformaldehyde in PBS at the indicated time points. Fixed tissues were paraffin embedded and stained with Hematoxylin and Eosin (H&E). One section was obtained per mouse and 3 livers were examined from each of the 2 independent experiments.

### Detection of DENV antigen in the liver

Liver tissue was snap-frozen in OCT compound (Tissue-Tek) prior to frozen-sectioning using a cryostat (Leica). Sections (10μm) were fixed onto slides at 4°C in 100% acetone, followed by blocking using 1% BSA in PBS. Tissue sections were then incubated in primary antibodies against CD11b (biotinylated), CD31 (both from eBiosciences), and dengue NS3 (GeneTex) overnight at 4°C. After washing, secondary antibodies including anti-rabbit AlexaFluor-594, streptavidin-conjugated Cy5 (both from Invitrogen), and anti-rat AlexaFlour-488 (Jackson ImmunoResearch) were incubated on the tissue sections in 1% BSA in PBS. Slides were washed and mounted using ProLong Gold Antifade reagent (ThermoFisher Scientific). Confocal images of stained tissue sections were obtained with Leica confocal laser scanning instrument with a channel-series approach to spectral overlap. Images were prepared for publication using ImageJ software.

### Hematology

Mouse blood samples were collected in K2EDTA and serum tubes (Greiner bio-one, Alphen a/d Rijin, The Netherlands). Whole blood was immediately analyzed for cell counts using automated hematology analyzer Cell Dyn– 3700 (Abbott Laboratories, MediSense Products, MA, USA). Serum aspartate (AST) transaminase levels were quantified using chemistry analyzer COBAS C111 (Roche, Basel, Switzerland).

### Assessment of vascular leakage

Vascular leakage was assessed using Evans Blue dye as a marker for albumin extravasation as described previously [27]. Briefly, the mice were injected with 50 μg/g body weight of Evans blue dye (0.5% in sterile phosphate-buffered saline, PBS) via the iv route. After 2 hours, the animals were euthanized and extensively perfused with PBS. The tissues were harvested and weighed prior to dye extraction using 4 ml/g wet tissue of N,N-dimethylformamide (Sigma) at 37°C for 24 h after which absorbance was read at 620 nm. Data were expressed as absolute absorbance at OD_620_ per gram of wet tissue. 5 mice per group per time point were used.

### Detection of cytokines and other soluble mediators

A 32-multiplex assay (Merck Millipore, Darmstadt, Germany) was used to measure different cytokine/chemokine in the mouse sera from three independent experiments. The following cytokines and chemokines were measured: Eotaxin, VEGF, TNF-α, RANTES, MIP-2, MIP-1β, MIP-1α, MIG, M-CSF, MCP-1, LIX, LIF, KC-like, IP-10, IL-17, IL-15, IL-13, IL-12 (p70), IL-12 (p40), IL-10, IL-9, IL-7, IL-6, IL-5, IL-4, IL-3, IL-2, IL-1β, IL-1α, IFN-γ, GM-CSF and G-CSF. The assay was performed according to the manufacturer’s instructions. The cytokine concentrations in DENV2-infected mice born to DENV1-immune mothers were then compared to those corresponding in the infected mice born to naïve mothers. Differentially expressed soluble mediators in this ADE context are identified as those (1) with levels in the infected mice born to DENV1-immune mothers that were statistically significant (*p* < 0.05 based on the Mann-Whitney test) as compared to that in the infected mice born to naïve mothers (for at least two out of three independent experiments) and (2) the levels in infected mice were significantly different from the basal levels in the uninfected controls (*p* < 0.05 based on Mann-Whitney test).

### *In vivo* cytokine neutralization

Five to six-week old A129 mice born to DENV1-immune mothers were infected with 10^6^ PFU of D2Y98P-PP1 via the iv route. At day 2 p.i., mice were injected iv with 100 μg of anti-TNFα (eBioscience, Cat. no. 167322–85), anti-IL6 (eBioscience, Cat. no. 16-7322-85), anti-IFNγ (eBioscience, Cat. no. 16-7311-85) or Rat IgG1 isotype control (eBioscience, Cat. no. 16-4301-85) per mouse.

## Supporting Information

S1 Fig Virus replication in 5–6 weeks old DENV1-infected A129 females. Five to six weeks old naïve female A129 mice were infected iv with 10^6^ PFU of DENV1 and bled at day 2, 4 and 6 p.i. for determination of viremia by plaque assay in BHK cells (A). At 6 weeks p.i., the mice were also bled and their anti-NS1 titers were determined by indirect ELISA against purified NS1 protein. Sera from age-matched naïve control mice were also included (** *p* < 0.01 based on Mann-Whitney test) (B).
(TIF)

S2 Fig Spleen, intestine and liver histology in DENV2-infected mice born to DENV1-immune mothers. 5-6-weeks old A129 mice born to either DENV1-immune or naïve mothers were iv infected with 10^6^ PFU of D2Y98P-PP1 (n = 3). Histological analysis of the liver harvested at day 2 p.i. and of the spleen and small intestine harvested at day 4 p.i. was performed. Images were taken at 5x (small intestines and spleen) or 20x (liver) magnification. Representative sections from two independent experiments are shown (scale bar– 100μm).
(TIF)

S3 Fig Blood parameters in DENV2-infected mice born to DENV1-immune or naïve mothers. Five to six-weeks old A129 mice born to either DENV1-immune (black bar) or naive mothers (open bar) were iv infected with 10^6^ PFU of D2Y98P-PP1, uninfected controls are depicted with a striped bar. At each of the indicated time points p.i., 5 mice per group were euthanized and blood was collected in EDTA-containing tubes for measurement of various blood parameters including white blood cells (WBC), neutrophils (NEU), lymphocytes (LYM), monocytes (MONO), hematocrit (HCT), and platelet (PLT) counts. * *p*<0.05, based on 1-way ANOVA with Bonferroni’s post-test.
(TIF)
