# Supplementary figures and images for: Maternal Antibody-Mediated Disease Enhancement in Type I Interferon-Deficient Mice Leads to Lethal Disease Associated with Liver Damage
Source: PLoS Negl Trop Dis. 2016 Mar 23;10(3):e0004536. doi: 10.1371/journal.pntd.0004536 (PMC4805191; doi:10.1371/journal.pntd.0004536)

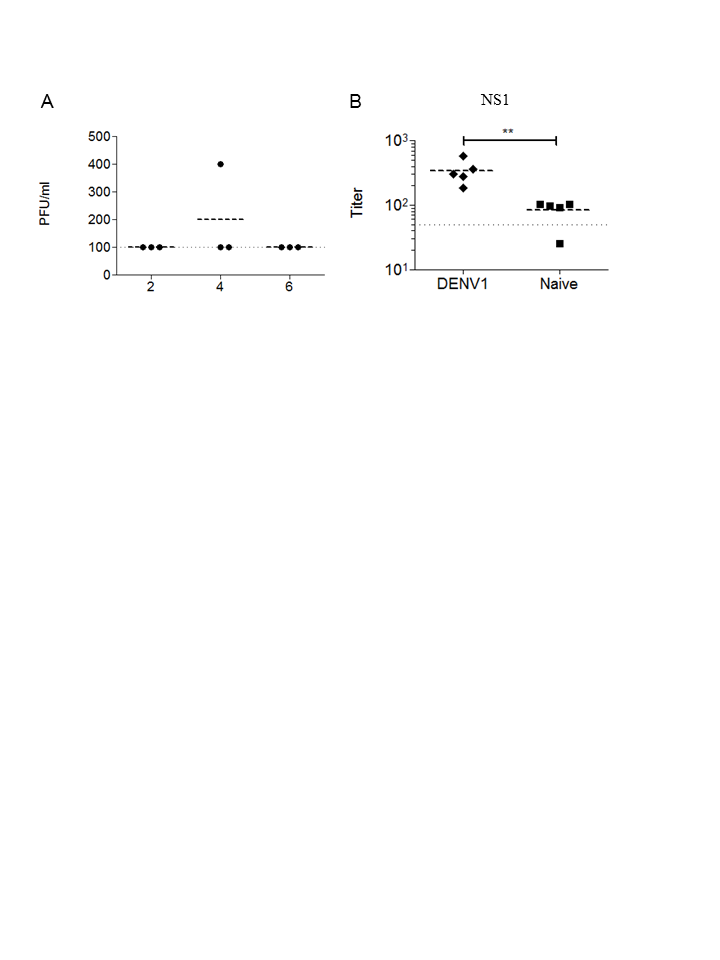

Supplement: S1 Fig — Five to six weeks old naïve female A129 mice were infected iv with 106 PFU of DENV1 and bled at day 2, 4 and 6 p.i. for determination of viremia by plaque assay in BHK cells (A). At 6 weeks p.i., the mice were also bled and their anti-NS1 titers were determined by indirect ELISA against purified NS1 protein. Sera from age-matched naïve control mice were also included (** p < 0.01 based on Mann-Whitney test) (B). (TIF) [file pntd.0004536.s001.tif]

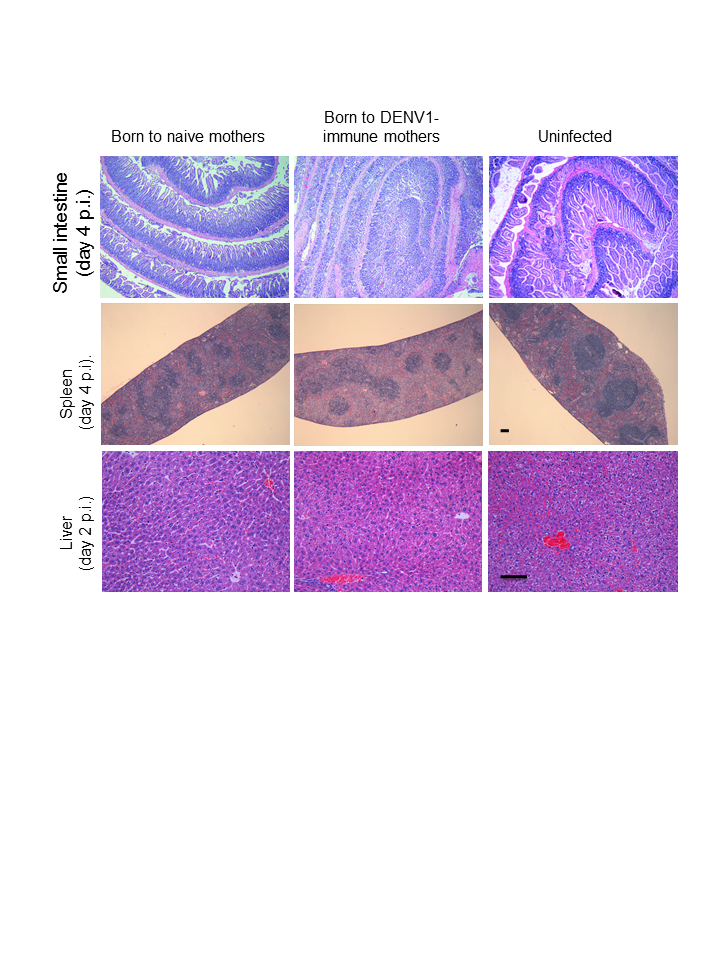

Supplement: S2 Fig — 5-6-weeks old A129 mice born to either DENV1-immune or naïve mothers were iv infected with 106 PFU of D2Y98P-PP1 (n = 3). Histological analysis of the liver harvested at day 2 p.i. and of the spleen and small intestine harvested at day 4 p.i. was performed. Images were taken at 5x (small intestines and spleen) or 20x (liver) magnification. Representative sections from two independent experiments are shown (scale bar– 100μm). (TIF) [file pntd.0004536.s002.tif]

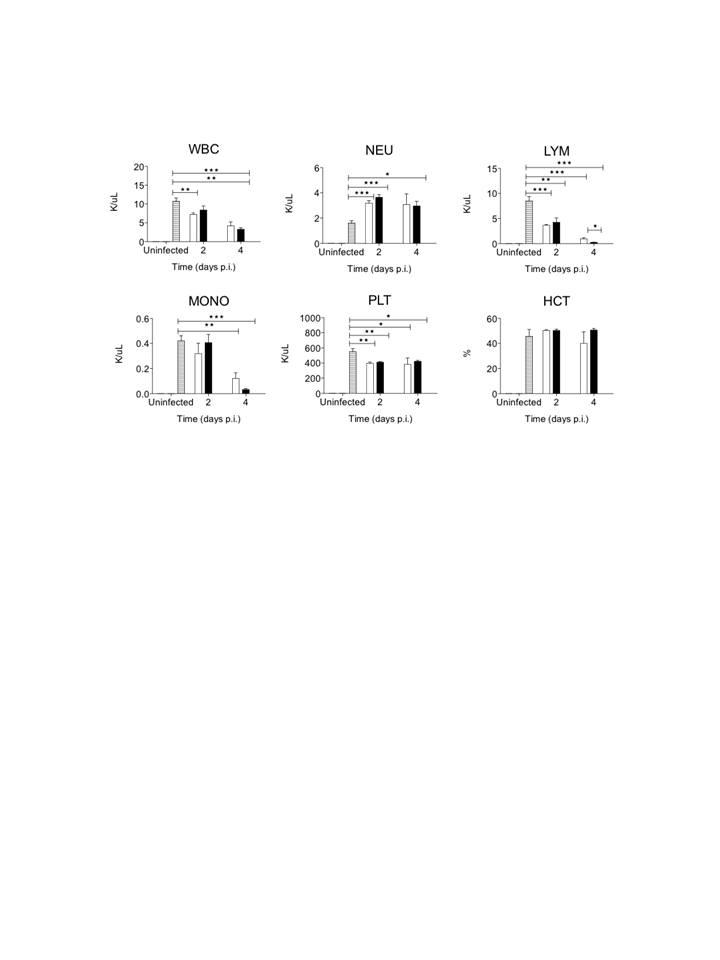

Supplement: S3 Fig — Five to six-weeks old A129 mice born to either DENV1-immune (black bar) or naive mothers (open bar) were iv infected with 106 PFU of D2Y98P-PP1, uninfected controls are depicted with a striped bar. At each of the indicated time points p.i., 5 mice per group were euthanized and blood was collected in EDTA-containing tubes for measurement of various blood parameters including white blood cells (WBC), neutrophils (NEU), lymphocytes (LYM), monocytes (MONO), hematocrit (HCT), and platelet (PLT) counts. * p<0.05, based on 1-way ANOVA with Bonferroni’s post-test. (TIF) [file pntd.0004536.s003.tif]
